# Spatio-temporal variation in bird assemblages is associated with fluctuations in temperature and precipitation along a tropical elevational gradient

**DOI:** 10.1371/journal.pone.0196179

**Published:** 2018-05-10

**Authors:** Vinicio Santillán, Marta Quitián, Boris A. Tinoco, Edwin Zárate, Matthias Schleuning, Katrin Böhning-Gaese, Eike Lena Neuschulz

**Affiliations:** 1 Senckenberg Biodiversity and Climate Research Centre Frankfurt, Frankfurt am Main, Germany; 2 Department of Biological Sciences, Goethe-Universität Frankfurt, Frankfurt am Main, Germany; 3 Escuela de Biología, Ecología y Gestión, Universidad del Azuay, Cuenca, Ecuador; U.S. Geological Survey, UNITED STATES

## Abstract

Understanding the spatial and temporal dynamics of species assemblages is a main challenge in ecology. The mechanisms that shape species assemblages and their temporal fluctuations along tropical elevational gradients are particularly poorly understood. Here, we examined the spatio-temporal dynamics of bird assemblages along an elevational gradient in Ecuador. We conducted bird point counts at three elevations (1000, 2000 and 3000 m) on 18 1-ha plots and repeated the sampling eight times over two years (216 hours in total). For each plot, we obtained data of monthly temperatures and precipitation and recorded the overall resource availability (i.e., the sum of flower, fruit, and invertebrate resources). As expected, bird richness decreased from low to high elevations. Moreover, we found a significant decrease in bird abundance and richness and an increase in evenness between the most and least humid season at each of the three elevations. Climatic factors were more closely related to these temporal fluctuations than local resource availability. While temperature had significant positive effects on the abundance of birds at mid and high elevations, precipitation negatively affected bird abundance at low and mid elevations. Our study highlights that bird assemblages along tropical elevational gradients can show pronounced seasonal fluctuations. In particular, low temperatures and high precipitation seem to impose important constraints on birds. We conclude that potential changes in climate, due to global warming, are likely to affect the spatio-temporal dynamics of bird assemblages along tropical elevational gradients.

## Introduction

Understanding the spatial and temporal patterns in species’ abundance and richness along environmental gradients is a fundamental challenge in ecology [1]. Many studies have shown that climate [2,3] and productivity [4,5] determine the structure of species assemblages across large spatial scales. However, the mechanisms that drive spatio-temporal dynamics of species assemblages have received little attention so far [6–8].

Elevational gradients present a great opportunity to study the spatial patterns of species assemblages because they comprise a variety of environmental conditions across relatively small spatial extents [9]. Many previous studies have, for instance, examined the spatial pattern in bird species richness along elevational gradients and showed that bird diversity generally declines with increasing elevation [10–13]. Climate has been identified as the main factor influencing bird assemblages along these gradients [14], in particular at high elevations where climatic conditions are harsh [15]. Climatic factors, such as temperature and precipitation, may affect birds directly via physiological constraints [16], for instance by restricting the activity, mobility and foraging time of birds [17]. Additionally, temperature and precipitation may also have indirect effects on birds via net primary productivity [18], which determines the amount of resources available to birds [19]. However, the degree to which primary productivity translates into a high diversity of birds strongly depends on the capacity of birds to obtain the available resources [20,21]. A previous study has shown that guild-specific resources, such as invertebrate biomass, can be more important determinants of the spatial richness patterns of avian feeding guilds than climatic factors [8]. The extent to which abiotic and biotic factors shape the spatial patterns of bird assemblages may vary across environmental gradients [9,14] and among spatial scales [22,23]. Under harsh environmental conditions, such as at high elevations, abiotic factors often determine the structure of bird assemblages [2,15]. Under benign environmental conditions (e.g., at low elevations), biotic factors, such as the competition for resources, may play a critical role in shaping bird assemblages [15,24]. Biotic factors are also expected to be more important, and better detectable, at small than at large spatial scales [23,25].

While the spatial pattern of bird species richness have been relatively well studied, temporal dynamics of bird assemblages are less known, specifically in tropical ecosystems [26] that are characterized by relatively constant climatic conditions throughout the year [27]. However, many tropical ecosystems are, in fact, characterized by seasonality, for instance by seasonal variation in precipitation [28,29]. Temporal changes in climatic conditions can occur locally, resulting in climatic variability [30] and in fluctuations in resource availability [31] on relatively small spatial scales. Only few studies so far have examined the temporal dynamics of tropical bird assemblages. These studies have shown pronounced temporal fluctuations of bird assemblages [32] and suggest that both changes in temperature and precipitation [33], as well as in resource availability [31] can cause local fluctuations of bird assemblages. However, none of these studies has simultaneously tested how climate factors and resource availability affect the spatial and temporal dynamics of bird assemblages across environmental gradients.

In this study, we examined the spatio-temporal dynamics of bird assemblages along an elevational gradient within and around Podocarpus National Park in Southern Ecuador. First, we tested the effects of elevation (i.e., 1000, 2000, and 3000 m) and season (most humid and least humid season) on bird abundance, evenness and richness. Second, we examined whether climate (i.e., temperature and precipitation) and/or resource availability (i.e., the sum of flower, fruit and invertebrate resources) explained the temporal fluctuations in bird abundance, evenness and richness along the elevational gradient. We hypothesized that 1) bird abundance, evenness and richness would decrease with increasing elevation [14,34] and 2) that the effect of seasonal variation in climate and resources on bird assemblages may vary across the three elevations [32], due to different constraints at high and low elevations. We expected that fluctuations of bird assemblages relate to both climatic factors and resource availability. While we expected that temperature and precipitation limit bird abundance, evenness and richness mostly at high elevations, likely due to physiological constraints [2,14], we expected resource availability to affect bird abundance, evenness and richness in particular at low elevations, due to high competition for resources [24].

## Material and methods

### Study area

**Fig 1:**
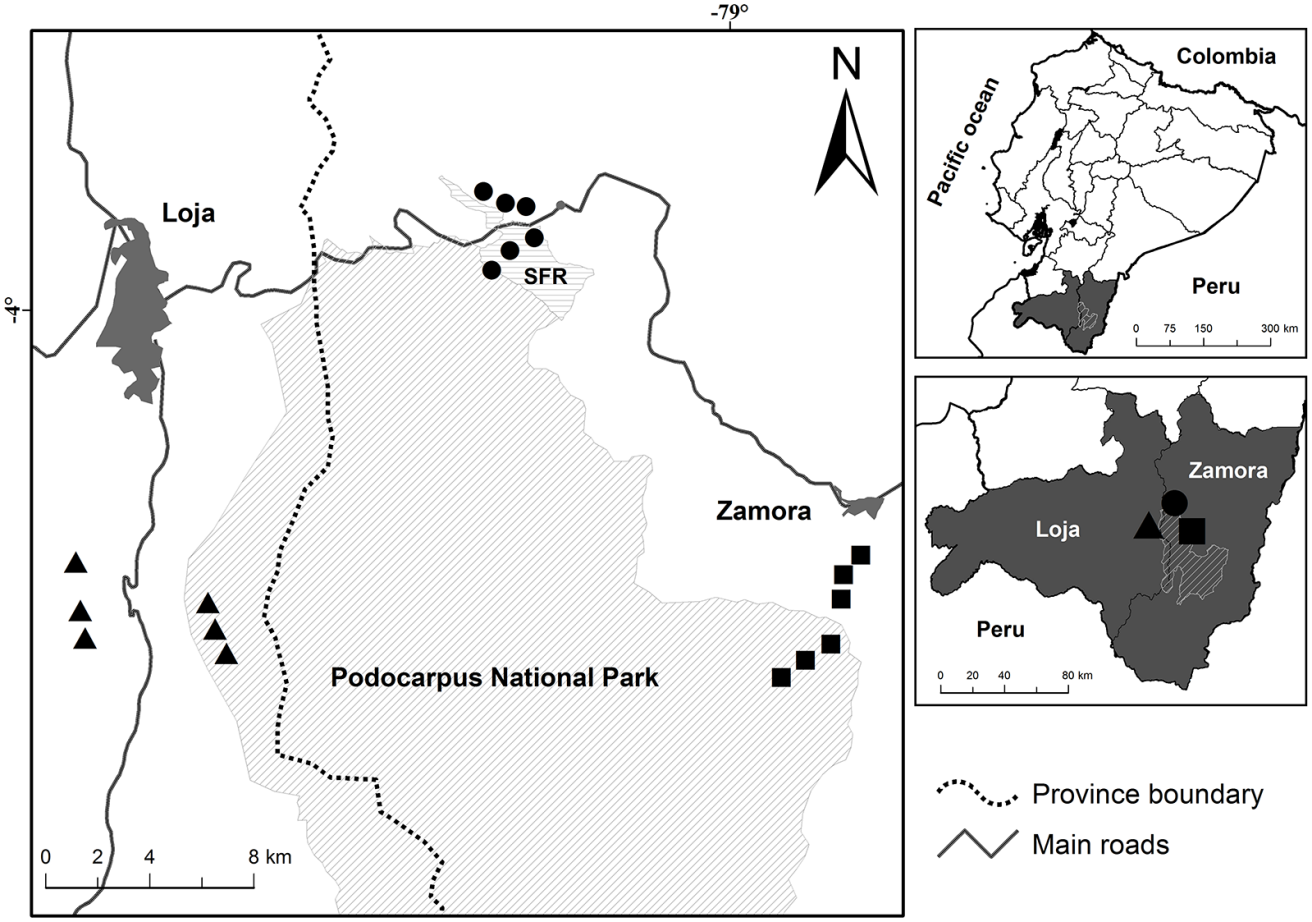
Study area within and around Podocarpus National Park and San Francisco reserve (SFR) in southern Ecuador. Squares represent study plots at 1000 m, circles those at 2000 m and triangles those at 3000 m.

We carried out this study within and around Podocarpus National Park and San Francisco reserve in southern Ecuador (Fig 1). The region is characterized by three vegetation types, evergreen premontane forest at low elevations (1000 m), evergreen lower montane forest at mid elevations (2000 m) and upper montane forest at high elevations (3000 m) [35]. The climate is tropical humid with a mean annual temperature of 20°C at low elevations, 15.5°C at mid elevations and 10°C at high elevations [28]. Mean annual precipitation is 2432 mm at low elevations, 2079 mm at mid elevations and 4522 mm at high elevations [28]. At each of three elevations, we selected two study sites. At each study site, we established three one-hectare plots, resulting in a total of 18 plots (Fig 1). Plot selection was conducted within the framework of the “Platform for Biodiversity and Ecosystem Monitoring and Research in South Ecuador”; the selected plots are representative for local site conditions.

### Bird point counts and surveys of resource availability

We conducted point counts in each of the 18 plots. Bird assemblages were sampled twice in the most humid season (May-July) and twice in the least humid season (September-November) in 2014 and 2015, resulting in eight temporal replicates per plot. At each plot, we placed nine point count locations, eight at the borders of the 1-ha plots and one in the centre. For 10 minutes, we recorded and identified all birds heard or seen to species level [36] within a 20-m radius around the centre of each point count location. The 20- metre sampling radius was chosen because of the low visibility in the dense tropical forest beyond that radius [8,37]. Sampling started at sunrise and ended before 09:00 h and was conducted by three observers. Plots were randomized among observers to minimize sampling bias. We quantified bird abundance, evenness and species richness by summing the records of all point counts per plot and temporal replicate (sampled area for each plot approx. 1.1 ha). Abundance was measured as the overall number of bird individuals per plot and temporal replicate. Evenness measures the relative abundance of each species in the community and was calculated as e ^(H)^ / S, where H is the Shannon diversity index and S the species richness per plot and temporal replicate [38]. Species richness was measured as the overall number of bird species recorded per plot and temporal replicate. We computed species accumulation curves on the relationship between the proportion of recorded species and the number of point counts conducted on each plot in each season (i.e., 18 point counts over both years). Similar slopes and saturating trends of the accumulation curves for the most humid and least humid season indicated that communities were similarly well sampled in both seasons (S1 Fig). On average, over 90% of the bird species were recorded after 13 point counts (S1 Fig). To further test whether bird detectability differed among elevations and between seasons, we recorded the distance of each bird from the centre of the point count location in all counts conducted in 2015. We found that the median distance of birds to the observer did not differ significantly among elevations (Generalized linear mixed effects model, low elevation compared to mid elevation: β = -0.18, z = -1.45, p = 0.15; low elevation compared to high elevation: β = -0.15, z = -1.25, p = 0.21) and between season (β = -0.16, z = -1.56, p = 0.12), indicating that the detectability of birds was similar across elevations and seasons.

We recorded the overall resource availability for each plot, comprising flower, fruit and invertebrate resources. To estimate flower and fruit availability, we recorded all plants with open flowers and ripe fruits within a 20-metre radius around each point count location. For each plant, we choose several randomly-picked branches, counted the number of flowers and fruits per branch and estimated the overall abundance of flowers and fruits per plant. Flower and fruit abundances of each of the nine point count locations were summed to obtain the overall abundance per plot [31]. To obtain a relative comparison of invertebrate resources among all plots, we assessed understory invertebrate biomass by using a standardized sweep-netting design [31]. We made a total of 100 sweeps along one of the 100-metre borders of each plot and subsequently weighted the cumulative invertebrate fresh biomass. Flowers, fruits and insect biomass were scaled to zero mean and unit variance and then summed to calculate the overall resource availability per plot.

### Data analyses

We used R v. 3.3.0 [39] for all statistical analyses. We obtained average monthly climate data for each study plot (S2 Fig). Average monthly within-forest temperatures (i.e., monthly mean of daily maximum temperatures) were obtained through an air temperature regionalization tool developed for the study region [40]. Monthly mean precipitation (i.e., average of the sum of monthly precipitation) was obtained through remote sensing techniques (local area weather radar and satellite imagery) and meteorological data [29]. Combining remote sensing techniques with meteorological data using geostatistical tools is most suitable to derive local climate information of high spatial and temporal resolution for the eastern slope of the southern Andes in Ecuador [40,41].

First, we tested the effect of elevation (three levels: 1000, 2000, 3000 m) and season (two levels: most humid and least humid season) on bird abundance, evenness and richness using generalized linear mixed effects models (GLMMs) assuming a Poisson error distribution for abundance and richness and a Gaussian error distribution for evenness. To account for the spatio-temporal sampling structure, we included the study plot nested in site and the sampling month in each respective year (i.e., in total eight sampling months over two years) as random effects in all models. We fitted all models with and without the interaction term between elevation and season and selected the best model based on the lowest Akaike’s information criterion [42]. We retained the simple model without interaction term in all cases (see S1 Table). To test whether patterns in bird richness were driven by patterns in abundance, we built a model of richness and included abundance as a predictor in addition to elevation and season.

Second, we tested whether temperature, precipitation and/or resource availability (i.e., the sum of flower, fruit, and invertebrate resources) explained temporal fluctuations in bird abundance, evenness and richness over the eight temporal point count replicates using GLMMs assuming a Poisson error distribution. We built separate models for each predictor variable and for each elevation (i.e., nine models in total). All predictor variables were scaled prior to the analyses to achieve comparability among models. We included the respective predictor variable as fixed effect and random intercept and slope effects of the study plot in all models allowing for potential differences in intercept and slopes among study plots. All models were fitted with a restricted maximum likelihood approach assuming a Poisson error distribution for abundance and richness and a Gaussian error distribution for evenness. To account for multiple testing across the nine models, we used a Bonferroni correction. To maintain a critical error rate of α = 0.05, we considered an effect significant if p < 0.005 [43].

To test whether different bird feeding guilds respond differently to their respective resource type, we classified the birds recorded per plot according their diet into nectarivores, frugivores, insectivores, and omnivores. We assigned birds consuming 60% or more of a food type (e.g. fruits) to the respective feeding guild (e.g. frugivores, see also Pigot et al. [44], S2 Table) based on the Elton trait database [45]. We then repeated the analyses and tested whether temperature, precipitation and/or the availability of the resource type (i.e., flowers, fruits, *or* invertebrates, respectively) explained temporal fluctuations in bird abundance separately for each of the feeding guilds.

## Results

We recorded 4323 individuals of 241 species across all elevations and seasons. Among these, 1589 individuals of 127 species were recorded at low elevations, 1494 individuals of 100 species at mid elevations and 1240 individuals of 70 species at high elevations. While 1694 individuals of 185 species were recorded in the most humid season, 2629 individuals of 208 species were recorded in the least humid season (see Table 1 for an overview of abundance and species richness of bird feeding guilds across all elevations and seasons).

**Table 1:** Overview of bird abundances and species richness belonging to different feeding guilds across all elevations in both study seasons. MHS = most humid season, LHS = least humid season, Ind = number of individuals, Spp = number of species.

	1000 m				2000 m				3000 m			
	MHS		LHS		MHS		LHS		MHS		LHS	
	Ind	Spp	Ind	Spp	Ind	Spp	Ind	Spp	Ind	Spp	Ind	Spp
Nectarivores	31	11	49	13	43	11	84	11	44	11	57	8
Frugivores	237	27	371	31	78	12	154	16	38	8	64	7
Insectivores	209	34	321	42	317	36	425	36	301	25	528	28
Omnivores	179	19	192	22	136	15	257	19	81	12	127	16
TOTAL	656	91	933	108	574	74	920	82	464	56	776	59

**Fig 2:**
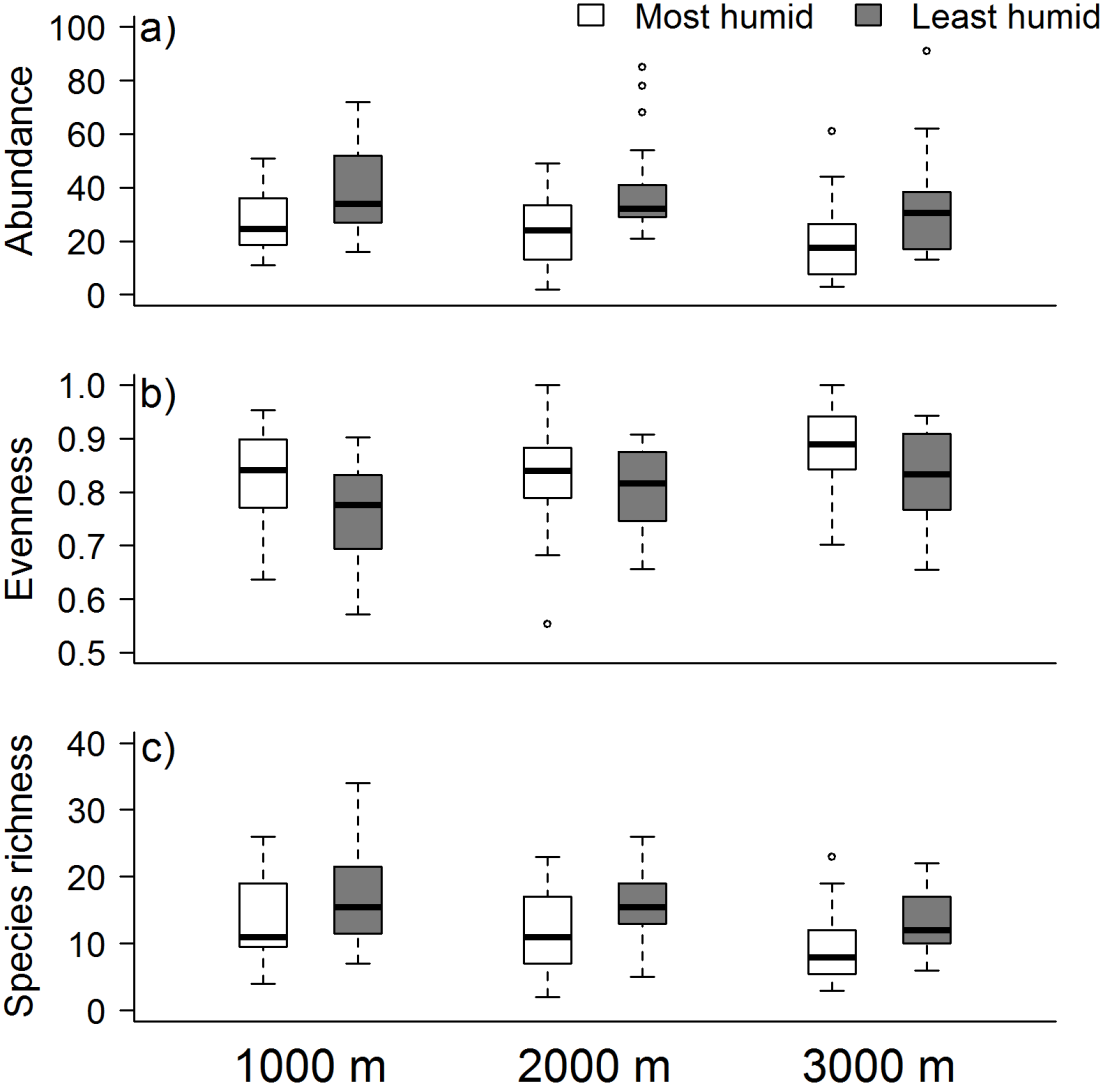
Spatio-temporal fluctuations of a) bird abundance, b) evenness and c) species richness across three elevations (1000, 2000, 3000 m) and in the most humid (white) and least humid (grey) season. Each box depicts the median, and 25th and 75th percentiles of bird records of six plots replicated four times within the respective season. Whiskers indicate the normal data range, circles represent outliers.

Bird abundance and richness were positively correlated (r = 0.73, p < 0.01), whereas abundance and richness were negatively related with evenness (r = -0.5, p < 0.001; r 0–0.13, p = 0.11, respectively). Bird abundance per plot was significantly lower in the most humid compared to the least humid season (most humid season: mean = 24, SD = 13.2, n = 72; least humid season: mean = 37, SD = 17, n = 72), but did not significantly differ among elevations (low elevation: mean = 33, SD = 14.9, n = 48; mid elevation: mean = 31, SD = 16.4, n = 48; high elevation: mean = 26, SD = 17.6, n = 48; Table 2a and S3 Table; Fig 2a). Bird evenness was significantly higher in the most humid compared to the least humid season (most humid season: mean = 0.844, SD = 0.093, n = 72; least humid season: mean = 0.797, SD = 0.086, n = 72), and increased significantly at the highest elevation (low elevation: mean = 0.794, SD = 0.095, n = 48; mid elevation: mean = 0.818, SD = 0.088, n = 48; high elevation: mean = 0.849, SD = 0.087, n = 48; Table 2b and S3 Table; Fig 2b). Bird species richness was significantly lower in the most humid than in the least humid season (most humid season: mean = 12, SD = 5.8, n = 72; least humid season: mean = 15, SD = 5.7, n = 72), and decreased significantly at the highest elevation (low elevation: mean = 15, SD = 6.5, n = 48; mid elevation: mean = 14, SD = 5.5, n = 48; high elevation: mean = 12, SD = 5.6, n = 48; Table 2c and S3 Table; Fig 2c). In the bird richness model that additionally included bird abundance as a predictor, bird richness was significantly positively related to bird abundance and decreased at the highest elevation, but was unaffected by season (Table 2d and S3 Table).

**Table 2:** Generalized linear mixed effects models testing a) bird abundance, b) evenness, c) species richness as a function of elevation (1000, 2000, 3000 m) and season (most humid and least humid), and d) species richness as a function of abundance, elevation and season. Study plot nested in site and sampling months of each year were included as random effects in all models. All models assume a Poisson error distribution. Significant effects (p < 0.05) are printed in bold.

	Predictor variable	β	SE	p
a) Bird abundance	Most humid season	-0.45	0.12	**<0.001**
	Mid elevation	-0.06	0.21	0.776
	High elevation	-0.31	0.21	0.141
b) Bird evenness	Most humid season	0.05	0.01	**0.001**
	Mid elevation	0.02	0.02	0.265
	High elevation	0.06	0.02	**0.018**
c) Bird richness	Most humid season	-0.28	0.09	**<0.001**
	Mid elevation	-0.11	0.13	0.411
	High elevation	-0.3	0.13	**0.024**
d) Bird richness	Abundance	0.27	0.02	**<0.001**
	Most humid season	-0.05	0.05	0.348
	Mid elevation	-0.08	0.08	0.283
	High elevation	-0.18	0.08	**0.024**

**Fig 3:**
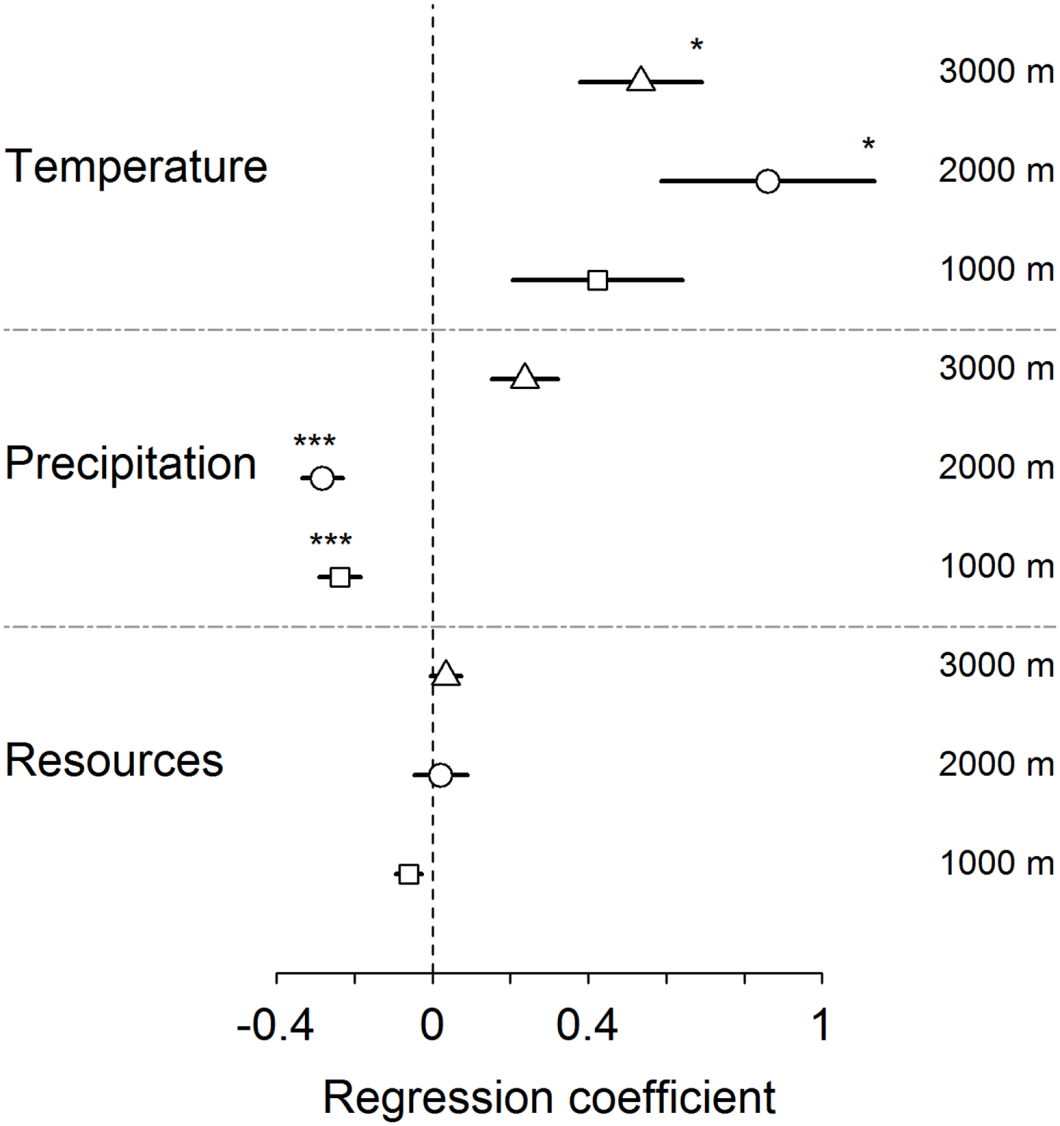
Effects on bird abundance of temperature, precipitation and resource availability on the temporal fluctuations along the elevational gradient. Squares represent sites at 1000 m, circles those at 2000 m, and triangles those at 3000 m. Shown are regression coefficients from generalized linear mixed effects models of eight temporal replicates including the respective predictor variable as fixed effect and random intercept and slope effects of the study plot in all models. Horizontal lines refer to standard error (SE). P-values after Bonferroni correction: *p<0.005, ***p<0.0001.

Climate factors were more important than resource availability for explaining the temporal fluctuations of birds along the elevational gradient (Table 3 and S4 Table; Fig 3). This pattern was only significant for bird abundance, albeit the patterns were similar for evenness and richness (Table 3 and S4 Table; Fig 3 and S3 Fig). Maximum temperature was positively related to bird abundance over the two study years and was significantly positively associated with bird abundance at mid and high elevations (Table 3a and S4 Table; Fig 3 and S3 Fig). In contrast, precipitation was negatively related to bird abundance over the two study years and was significantly negatively associated with bird abundance at low and mid elevations (Table 3a and S4 Table; Fig 3 and S3 Fig). Overall resource availability had no significant effect on the temporal variation in bird abundance, evenness and richness (Table 3 and S4 Table, Fig 3 and S3 Fig). Separate analyses for the different feeding guilds (nectarivores, frugivores, insectivores and omnivores) supported the pattern that climatic factors were generally more important in explaining temporal variation in these groups than their respective resource type (i.e., flowers, fruits, insects and all resources combined, S4 Fig).

**Table 3:** Generalized linear mixed effects models testing the effects of temperature, precipitation and resource availability on eight temporal replicate counts in a) bird abundance b) species evenness and c) species richness at three elevations. Estimates for each predictor variable and elevation result from separate models and assume a Poisson error distribution; all predictors were scaled to zero mean and unit variance prior to model fitting. All models include the respective predictor variable as fixed effect and random intercept and slope effects of the study plot. Significant effects after Bonferroni correction (p < 0.005) are printed in bold.

	Predictor variable	Elevation (m)	β	SE	p
a) Bird abundance	Temperature	3000	0.53	0.16	**0.001**
		2000	0.86	0.27	**0.002**
		1000	0.42	0.22	0.052
	Precipitation	3000	0.24	0.09	0.005
		2000	-0.28	0.05	**<0.001**
		1000	-0.24	0.05	**<0.001**
	Resources	3000	0.03	0.04	0.391
		2000	0.02	0.07	0.753
		1000	-0.06	0.03	0.079
b) Bird evenness	Temperature	3000	-0.06	0.03	0.072
		2000	-0.01	0.08	0.882
		1000	-0.13	0.05	0.013
	Precipitation	3000	0.004	0.02	0.861
		2000	0.01	0.01	0.542
		1000	0.04	0.02	0.104
	Resources	3000	-0.01	0.01	0.351
		2000	0.001	0.01	0.932
		1000	-0.02	0.01	0.288
c) Bird richness	Temperature	3000	0.38	0.21	0.078
		2000	0.61	0.39	0.116
		1000	0.15	0.2	0.464
	Precipitation	3000	0.12	0.09	0.21
		2000	-0.17	0.08	0.034
		1000	-0.14	0.08	0.073
	Resources	3000	-0.02	0.04	0.604
		2000	-0.09	0.03	0.005
		1000	-0.07	0.03	0.036

## Discussion

We show that bird species richness decreased significantly at high elevations and that bird abundance, evenness and richness varied significantly between the most humid and least humid season across all elevations. The pronounced temporal fluctuations in bird abundances were mainly related to climatic factors (i.e., temperature and precipitation) rather than by resource availability. Our findings suggest that the temporal fluctuations in tropical bird assemblages in our study region likely occur due to temporary constraints related to climatic conditions rather than due to resource limitations.

We found a significant decline of bird richness at the highest elevation, which even persisted when accounting for declines in bird abundance. Our results are in line with previous studies showing a decline of species richness along elevational gradients [14], which has been attributed to limiting abiotic and biotic factors, such as harsh climatic conditions or reduced resource availability at high elevations [18]. In contrast, we found no significant changes in overall bird abundance across the elevational gradient. In species-poor assemblages, such as at high elevations, the relative abundance of individual species is often higher compared to species-rich assemblages [34], which is consistent with the slight increase in species evenness at the highest elevations. Such effects of density compensation of the persisting species may explain similar overall bird abundance at all elevations.

While we did not find spatial patterns in bird abundances, we found pronounced temporal fluctuations. At all three elevations, bird abundances increased in the least humid season. This increase in abundance corresponded to a consistent decline of bird evenness in the least humid season, indicating a more skewed abundance distribution during that time, likely due to an increase in abundance of the dominant species in the assemblage. We also encountered changes in species richness between seasons, but these changes were largely driven by changes in bird abundance, as abundance changes accounted for the seasonal variation in bird richness (Table 2d). One explanation for seasonal fluctuations in bird abundances might be the narrow thermal tolerance of tropical species [26] that may force birds to leave their habitat if climatic conditions become temporarily unsuitable [46]. The consistent increase in bird abundances in the least humid season across all three elevations suggests medium- to long-distance seasonal movements of birds [47] rather than to short-distance elevational migrations among the low, mid and high elevation sites [33]. Another explanation could be differences in the detectability of birds across the course of the year. For instance, are vocally more active and visible during the breeding season [33]. However, breeding cycles of tropical birds are known to lack a pronounced seasonality and may differ between species of a local assemblage [46]. Moreover, both species accumulation curves and distance-sampling revealed no significant differences in bird detectability between seasons, suggesting that bird abundances and evenness indeed fluctuated strongly between seasons independent of bird activity.

In our study, temperature and precipitation had contrasting effects on the temporal fluctuation of bird abundance along the elevational gradient. Temperature had a significant positive effect on bird abundance at mid and high elevations, while precipitation had a significant negative effect at mid and low elevations. Our results are supported by previous studies showing that bird assemblages of low and high elevations may be affected by different climatic factors [14,48], probably due to specific physiological constrains under the respective climate conditions [17,49]. In fact, temperature and precipitation covered distinct extremes along the elevational gradient. For example, monthly maximum temperatures were much lower and more variable at high compared to low elevations (3000 m: mean = 13.1 °C, SD = 1.7; 1000 m: mean = 23.1 °C, SD = 1.2). While mean monthly precipitation was low at low elevations (mean = 188 mm, SD = 29), precipitation increased towards high elevations (mean = 213 mm, SD = 36). Further, high elevation forests in the study area are characterized by persistent cloud cover and fog all year long, resulting in additional moisture bound to aerosols [28,50]. While bird abundances were clearly affected by the low temperatures at high elevations, the high amount of rainfall did not seem to affect bird abundance. This pattern suggests that bird assemblages at high elevations are limited by temperature, but might be adapted to the persisting rainy conditions at these sites. In contrast, lowland bird assemblages are not limited by extreme temperatures, but rainfall may pose limitations to birds forcing them to leave the area [17,30]. The significant negative effect of precipitation on bird assemblages at low elevations conflicts our initial expectation that abiotic factors are the main constraints of bird assemblages only at high elevations [2,14]. In fact, most studies that have identified precipitation as a main predictor of bird assemblages demonstrate that high precipitation at upper elevations may cause down-slope movements of birds [17,30,51]. Interestingly, in our study, the overall amount of rainfall was comparatively moderate at low, compared to high elevations, but still significantly affected lowland bird assemblages. Other studies, mostly from water-limited ecosystems, have in turn shown positive effects of precipitation on bird assemblages [14]. Our results highlight that beside the well-studied negative effects of low temperatures [9,14], an excess of precipitation can lead to reduced abundances in bird assemblages.

In contrast to the significant effects of climatic factors, food resource availability did not contribute to explaining the temporal fluctuations in bird assemblages. Our findings are different to those of previous studies where resource availability influenced temporal variation of bird assemblages [31,32,52,53]. One explanation for this difference could be that most of these previous studies focused on particular species or feeding guilds rather than on the response of the entire bird assemblage to resource availability [4]. Separate analyses of different feeding guilds and their respective food resources, however, supported the pattern that climate rather than the availability of resources was more closely associated with temporal variation in bird guilds (S4 Fig). Another explanation for the low importance of resources for the spatio-temporal dynamics of bird assemblages could be the overall high productivity of the studied ecosystem [35,54]. In systems that provide a surplus of resources to animal consumers, such as birds, this could result in a decoupling of resource availability and consumer diversity [55]. However, resource effects on bird assemblages may generally be difficult to detect because the sampling of resources in tropical forests can never be exhaustive. In our study, we did, for instance, not account for invertebrates occurring in higher forest strata or the amount of nectar produced by flowers. Moreover, the local heterogeneity of resources was probably higher than that of temperature and precipitation, which could have contributed to the stronger relationship of bird abundance with climatic conditions than with resource availability. We therefore concede that resource effects on temporal fluctuations in bird abundance could be underestimated due to methodological constraints.

## Conclusions

In our study we showed that bird assemblages along an elevational gradient in the tropical Andes experienced strong seasonal variation that was governed by changes in temperature and precipitation. In particular, low temperature and high precipitation caused decreases in bird abundances. Although climatic factors are expected to increase in importance, relative to biotic factors, at large spatial scales [23], we show here that climatic constraints can overrule biotic effects at small spatial scales. The high importance of climatic factors in shaping the spatio-temporal dynamics of bird assemblages highlights the sensitivity of tropical birds towards projected climate change [56]. Climate change projections for the tropical Andes predict an increase of temperature, especially at high elevations, and an increase of extreme rainfall events, in particular at low elevations [57]. While bird species at high elevations might benefit from warmer temperatures, extreme drought events could also negatively affect high-elevation assemblages [58]. In the lowlands, projected increases in rainfall and in the temporal variation in precipitation will likely have negative effects on bird assemblages and could trigger an increase in spatio-temporal movements of lowland species in the future [58]. We conclude that understanding the spatio-temporal dynamics of species assemblages in response to shifts in temperature and precipitation are essential for projecting potential responses of species to future climatic conditions.

## Supporting information

S1 Fig Species accumulation curves. (PDF)

S2 Fig Climate data of the study region. (PDF)

S3 Fig Effects of temperature, precipitation and resource availability on the temporal fluctuations in bird evenness and bird species richness. (PDF)

S4 Fig Effects of temperature, precipitation and resource type on the temporal fluctuations in abundance of feeding guilds. (PDF)

S1 Table Akaike’s information criterion of models testing main and interaction effects of elevation and season on bird abundance, evenness and richness. (PDF)

S2 Table List of 241 bird species recorded and their feeding guilds. (PDF)

S3 Table Estimates of random effects for models testing the effects of elevation and season on bird communities. (PDF)

S4 Table Estimates of random effects for models testing the effects of temperature, precipitation and resource availability on the temporal fluctuations in bird communities. (PDF)
