# Supplementary material for: Spatio-temporal variation in bird assemblages is associated with fluctuations in temperature and precipitation along a tropical elevational gradient
Source: PLoS One. 2018 May 10;13(5):e0196179. doi: 10.1371/journal.pone.0196179 (PMC5945003; doi:10.1371/journal.pone.0196179)
Supplement: S1 Fig — (PDF) [file pone.0196179.s001.pdf]

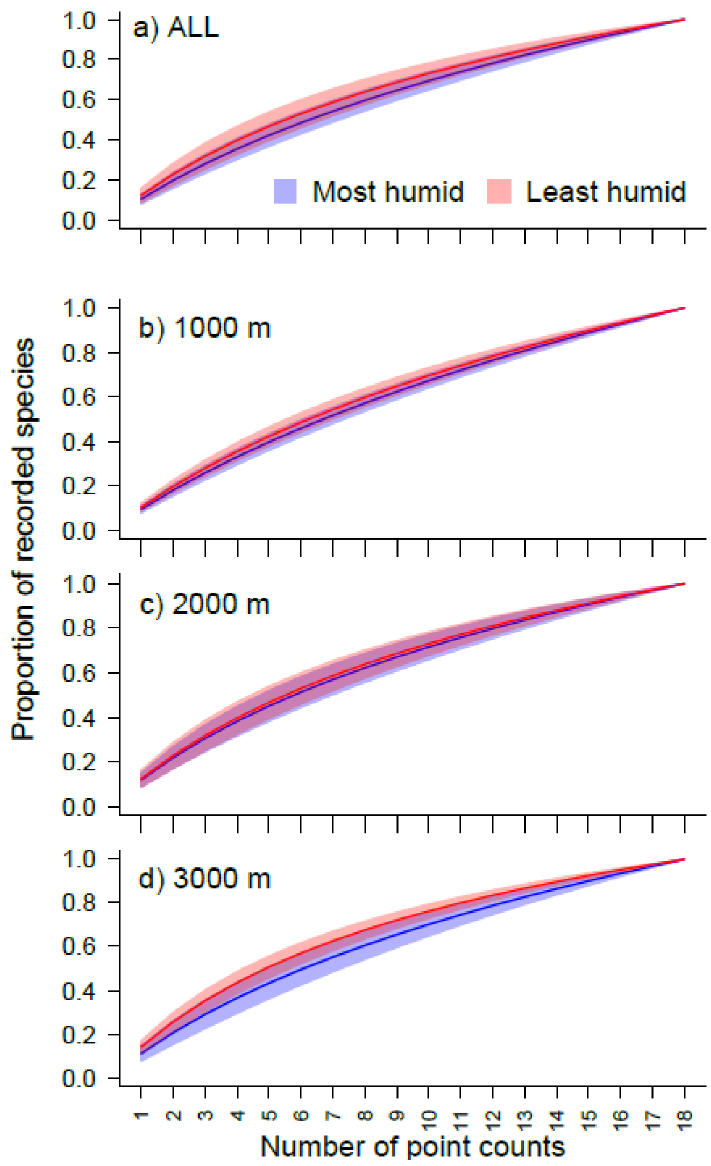

S1 Fig. Species accumulation curves showing the relationship between the proportion of recorded species and the number of point counts conducted on each plot in each season (i.e., 18 point counts in total over both years)**.** Curves were calculated for each plot and were averaged for (a) all study sites and (b, c, d) for each elevation separately. Blue lines represent mean species accumulation for the most humid season, red lines represent mean species accumulation for the least humid season. Blue and red areas show standard deviation for most humid and least humid seasons, respectively.
