# Supplementary material for: Spatio-temporal variation in bird assemblages is associated with fluctuations in temperature and precipitation along a tropical elevational gradient
Source: PLoS One. 2018 May 10;13(5):e0196179. doi: 10.1371/journal.pone.0196179 (PMC5945003; doi:10.1371/journal.pone.0196179)
Supplement: S2 Fig — (PDF) [file pone.0196179.s002.pdf]

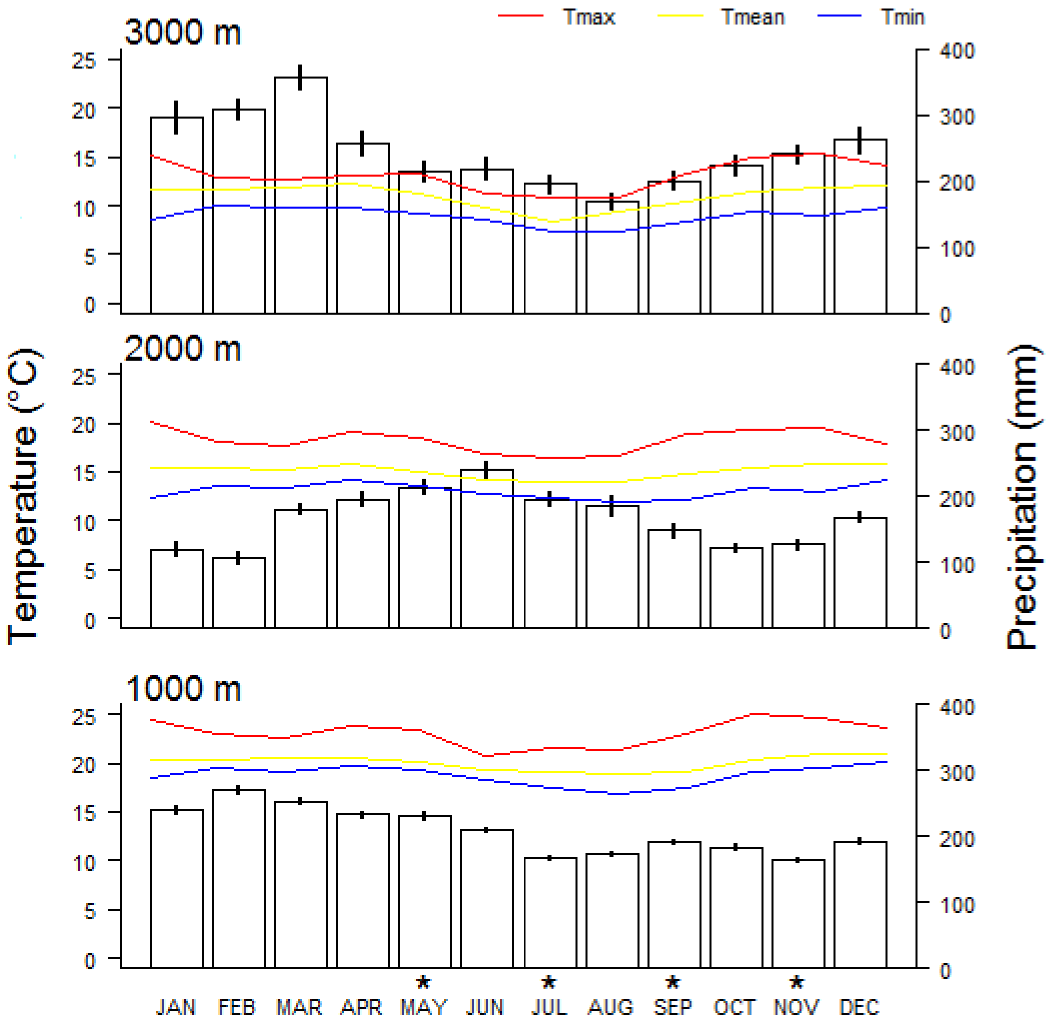

S2 Fig. Mean monthly temperature (i.e., monthly mean of daily minimum [blue], mean [yellow] and maximum [maximum] temperatures) and precipitation (i.e., average of the sum of monthly precipitation) over all six study plots located at 1000, 2000 and 3000 m a.s.l. Sampling months are indicated by an asterisk.
