# Supplementary material for: Spatio-temporal variation in bird assemblages is associated with fluctuations in temperature and precipitation along a tropical elevational gradient
Source: PLoS One. 2018 May 10;13(5):e0196179. doi: 10.1371/journal.pone.0196179 (PMC5945003; doi:10.1371/journal.pone.0196179)
Supplement: S3 Fig — (PDF) [file pone.0196179.s003.pdf]

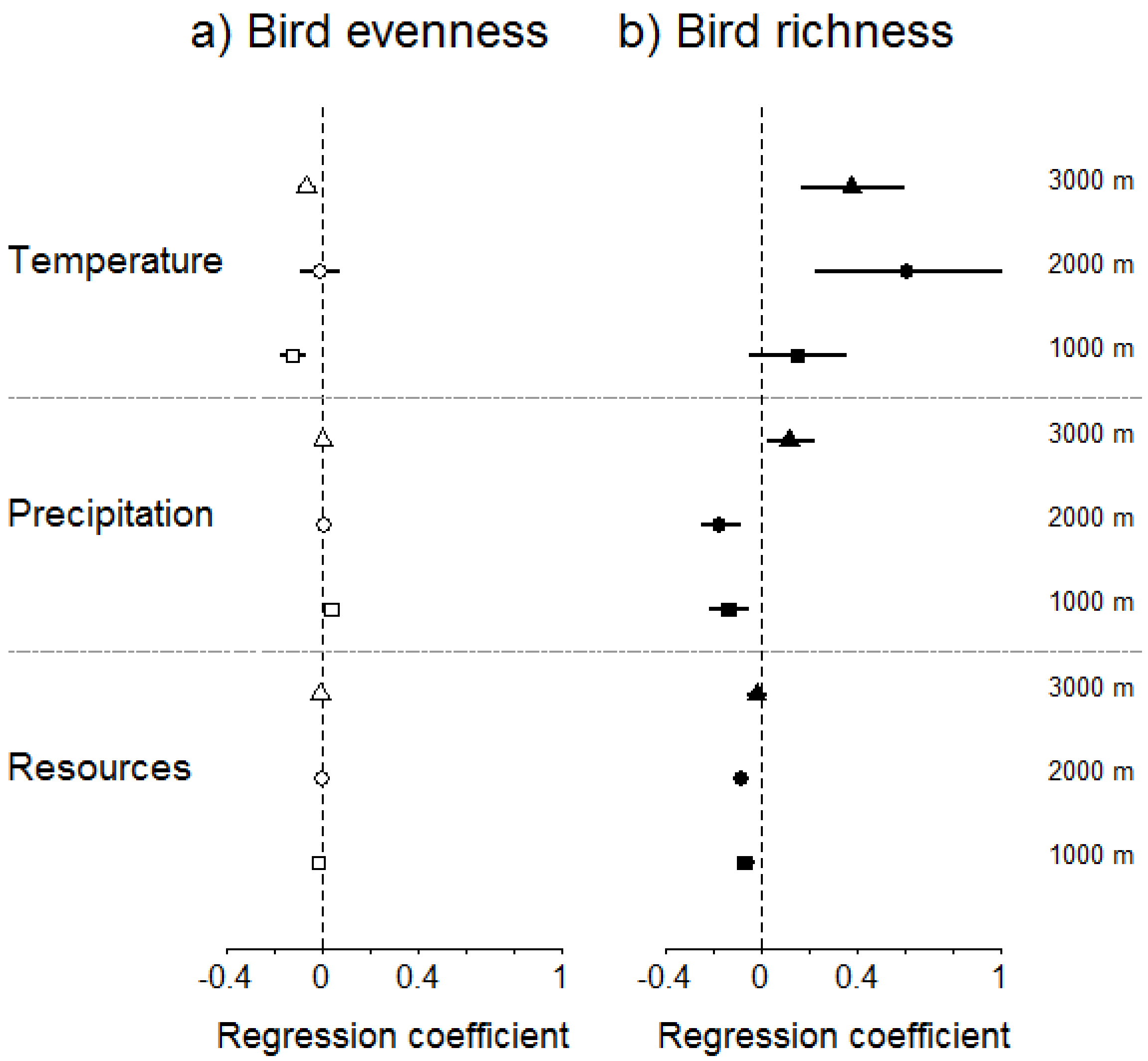

S3 Fig. Effects of temperature, precipitation and resource availability on the temporal fluctuations in a) bird evenness (white) and bird species richness (black) along the elevational gradient. Squares represent sites at 1000 m, circles those at 2000 m, and triangles those at 3000 m. Shown are regression coefficients from generalized linear mixed effects models of eight temporal replicates including the respective predictor variable as fixed effect and random intercept and slope effects of the study plot in all models. Horizontal lines refer to standard error (SE).
