# Supplementary material for: Spatio-temporal variation in bird assemblages is associated with fluctuations in temperature and precipitation along a tropical elevational gradient
Source: PLoS One. 2018 May 10;13(5):e0196179. doi: 10.1371/journal.pone.0196179 (PMC5945003; doi:10.1371/journal.pone.0196179)
Supplement: S4 Fig — (PDF) [file pone.0196179.s004.pdf]

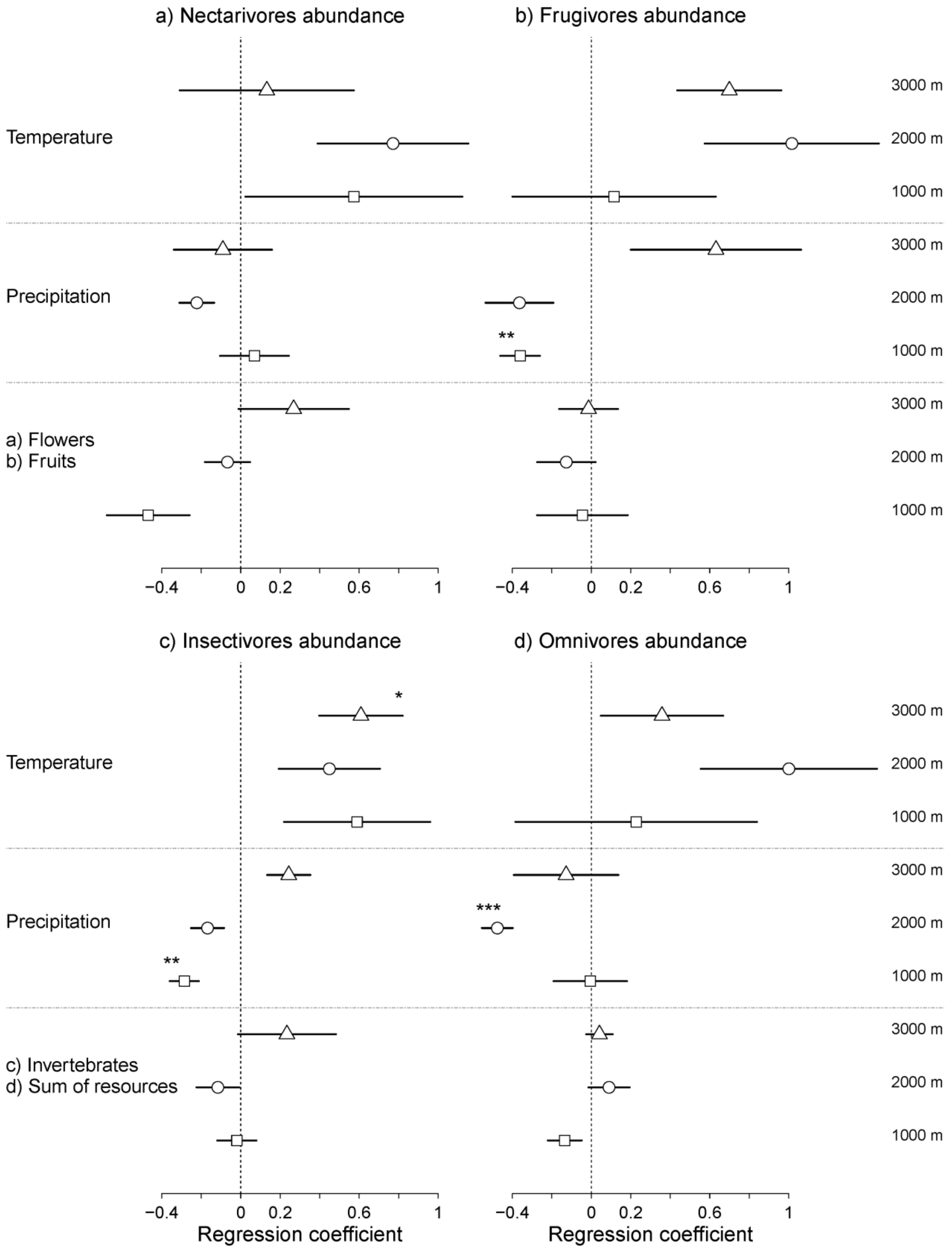
a) Nectarivores abundance
b) Frugivores abundance
Temperature
Precipitation
a) Flowers
b) Fruits
3000 m
2000 m
1000 m
**
−0.4
0
0.2
0.6
1
c) Insectivores abundance
d) Omnivores abundance
*
***
**
c) Invertebrates
d) Sum of resources
Regression coefficient

S4 Fig. Effects of temperature, precipitation and resource type [i.e., a) flowers, b) fruit, c) insects, d) sum of all resources] on the temporal fluctuations in abundance of a) nectarivores, b) frugivores, c) insectivores and d) omnivores along the elevational gradient. Squares represent sites at 1000 m, circles those at 2000 m, and triangles those at 3000 m. Shown are regression coefficients from generalized linear mixed effects models of eight temporal replicates including the respective predictor variable as fixed effect and random intercept and slope effects of the study plot in all models. Horizontal lines refer to standard error (SE). P-values after Bonferroni correction: *p<0.005, ***p<0.0001.
