# Supplementary material for: Spatio-temporal variation in bird assemblages is associated with fluctuations in temperature and precipitation along a tropical elevational gradient
Source: PLoS One. 2018 May 10;13(5):e0196179. doi: 10.1371/journal.pone.0196179 (PMC5945003; doi:10.1371/journal.pone.0196179)
Supplement: S1 Table — (PDF) [file pone.0196179.s005.pdf]

S1 Table. Akaike's information criterion (AIC) of generalized linear mixed effect models testing main and interaction effects of elevation and season on bird a) abundance, b) evenness, c) species richness. Species richness model in d) also includes abundance as a fixed effect. Study plot nested in site and sampling month of each year were included as random effects in all models. All models assume a Poisson error distribution.

	**Predictor variables**	**AIC**	**ΔAIC**
**a) Bird abundance**	Season + elevation	1391	1
	Season x elevation	1390	
**b) Bird evenness**	Season + elevation	-283	4
	Season x elevation	-279	
**c) Bird richness**	Season + elevation	927	3
	Season x elevation	930	
**d) Bird richness**	Abundance + season + elevation	833	4
	Abundance + season x elevation	837	
