# Supplementary material for: Spatio-temporal variation in bird assemblages is associated with fluctuations in temperature and precipitation along a tropical elevational gradient
Source: PLoS One. 2018 May 10;13(5):e0196179. doi: 10.1371/journal.pone.0196179 (PMC5945003; doi:10.1371/journal.pone.0196179)
Supplement: S2 Table — (PDF) [file pone.0196179.s006.pdf]

S2 Table. List of 241 bird species recorded and their feeding guilds (60% or more of a food type) based on the Elton trait database, at three elevations (1000, 2000 and 3000 m) in and around Podocarpus National Park and San Francisco reserve in southern Ecuador.

	Nectarivores	Frugivores	Insectivores	Omnivores
**1000**				
**Tinamiformes**				
Crypturellus soui				X
Tinamus tao				X
**Galliformes**				
Aburria aburri		X		
Chamaepetes goudotii		X		
Odontophorus speciosus				X
Ortalis guttata		X		
**Columbiformes**				
Columba plumbea				X
Columba subvinacea		X		
Geotrygon frenata				X
Leptotila rufaxilla		X		
**Psittaciformes**				
Pyrrhura albipectus		X		
**Cuculiformes**				
Crotophaga ani				X
Piaya cayana			X	
**Apodiformes**				
Aglaiocercus kingi	X			
Amazilia fimbriata	X			
Chrysuronia oenone	X			
Colibri coruscans	X			
Doryfera ludovicae	X			
Eutoxeres aquila	X			
Heliodoxa leadbeateri	X			
Heliothryx aurita	X			
Klais guimeti	X			
Ocreatus underwoodii	X			
Phaethornis griseogularis	X			
Phaethornis guy	X			
Phaethornis syrmatophorus	X			
Thalurania furcata	X			

**Trogoniformes**				
Trogon collaris			X	
**Coraciiformes**				
Momotus aequatorialis			X	
**Piciformes**				
Aulacorhynchus derbianus				X
Dryocopus lineatus			X	
Eubucco bourcierii		X		
Galbula pastazae			X	
Malacoptila fulvogularis			X	
Piculus rubiginosus			X	
Picumus lafresnayi			X	
Veniliornis passerinus			X	
**Passeriformes**				
Ammodramus aurifrons		X		
Ampelioides tschudii				X
Anabacerthia striaticollis			X	
Arremon aurantiirostris				X
Basileuterus fulvicauda			X	
Cacicus uropygialis			X	
Campylorhamphus pusillus			X	
Catharus ustulatus			X	
Cephalopterus ornatus				X
Cercomacra nigrescens			X	
Chamaeza campanisona			X	
Chlorochrysa calliparaea		X		
Chlorophanes spiza		X		
Chlorospingus canigularis				X
Chlorospingus flavigularis		X		
Cissopis leveriana		X		
Coereba flaveola	X			
Colonia colonus			X	
Conopias cinchoneti				X
Coryphospingus cucullatus				X
Cyanocorax violaceus				X
Cyanocorax yncas				X
Dacnis cayana				X
Dacnis lineata		X		
Dendrocincla fuliginosa			X	
Dixiphia pipra		X		
Dysithamnus mentalis			X	

Euphonia xanthogaster	X		
Glyphorynchus spirurus		X	
Grallaria haplonota		X	
Hemithraupis guira		X	
Henicorhina leucosticta		X	
Herpsilochmus axillaris		X	
Hylophilus olivaceus		X	
Hylophylax poecilinota		X	
Hypocnemis cantator		X	
Iridophanes pulcherrima	X		
Lepidothrix isidorei	X		
Leptopogon rufipectus		X	
Leptopogon superciliaris		X	
Lochmias nematura		X	
Machaeropterus striolatus	X		
Mionectes oleagineus	X		
Mionectes olivaceus	X		
Mionectes striaticollis			X
Myadestes ralloides			X
Myiarchus cephalotes		X	
Myiarchus ferox			X
Myioborus miniatus		X	
Myiotriccus ornatus		X	
Myiozetetes similis			X
Odontorchilus branickii		X	
Parula pitiayumi		X	
Phyllomyias plumbeiceps			X
Pipra erythrocephala	X		
Pipreola chlorolepidota	X		
Piranga leucoptera			X
Platycichla leucops	X		
Platyrinchus mystaceus		X	
Pogonotriccus ophthalmicus		X	
Pogonotriccus poecilotis		X	
Psarocolius angustifrons			X
Psarocolius decumanus			X
Rupicola peruviana	X		
Saltator grossus		X	
Saltator maximus		X	
Sittasomus griseicapillus		X	
Syndactyla subalaris		X	

Tachyphonus cristatus				X
Tangara arthus		X		
Tangara chilensis		X		
Tangara chrysotis		X		
Tangara cyanicollis		X		
Tangara gyrola		X		
Tangara nigrocincta		X		
Tangara punctata		X		
Tangara schrankii		X		
Tangara xanthogastra				X
Thraupis episcopus				X
Thraupis palmarum		X		
Todirostrum cinereum			X	
Tolmomyias viridiceps			X	
Troglodytes aedon			X	
Turdus albicollis			X	
Turdus fulviventris		X		
Turdus nigriceps		X		
Tyrannus melancholicus			X	
Wilsonia canadensis			X	
Xenops minutus			X	
Xiphorhynchus triangularis			X	
**2000**				
**Tinamiformes**				
Nothocercus bonapartei				X
**Galliformes**				
Chamaepetes goudotii		X		
Odontophorus speciosus				X
Penelope barbata		X		
**Columbiformes**				
Columba fasciata		X		
Geotrygon frenata				X
**Coraciiformes**				
Momotus aequatorialis			X	
**Psittaciformes**				
Amazona mercenaria		X		
Touit stictoptera				X
**Apodiformes**				
Adelomyia melanogenys	X			
Aglaiocercus kingi	X			
Chalcostigma ruficeps	X			

Coeligena coeligena	X			
Coeligena torquata	X			
Colibri coruscans	X			
Colibri thalassinus	X			
Doryfera johannae	X			
Doryfera ludovicae	X			
Heliangelus amethysticollis	X			
Heliodoxa leadbeateri	X			
Heliodoxa rubinoides	X			
Metallura tyrianthina	X			
Ocreatus underwoodii	X			
Phaethornis syrmatophorus	X			
**Trogoniformes**				
Pharomachrus auriceps		X		
Trogon personatus				X
**Piciformes**				
Aulacorhynchus prasinus				X
Campephilus pollens			X	
Piculus rivolii			X	
**Passeriformes**				
Anairetes parulus			X	
Anisognathus lacrymosus		X		
Anisognathus somptuosus				X
Atlapetes latinuchus				X
Basileuterus coronatus			X	
Basileuterus nigrocristatus			X	
Basileuterus tristriatus			X	
Buarremon brunneinuchus				X
Buarremon torquatus				X
Cacicus uropygialis			X	
Chlorospingus canigularis				X
Chlorospingus flavigularis		X		
Chlorospingus ophthalmicus			X	
Contopus fumigatus			X	
Creurgops verticalis			X	
Cyanocorax yncas				X
Cyclarhis gujanensis			X	
Dendroica fusca			X	
Diglossa albilatera				X
Diglossa humeralis				X
Diglossopis cyanea			X	

Drymophila caudata		X	
Elaenia albiceps			X
Elaenia pallatangae			X
Grallaria ruficapilla		X	
Grallaricula flavirostris		X	
Hemispingus frontalis		X	
Henicorhina leucophrys		X	
Iridosornis analis			X
Lepidocolaptes lacrymiger		X	
Leptopogon rufipectus		X	
Lochmias nematura		X	
Mecocerculus calopterus		X	
Mionectes olivaceus	X		
Mionectes striaticollis			X
Myadestes ralloides			X
Myiarchus cephalotes		X	
Myiarchus tuberculifer		X	
Myioborus miniatus		X	
Ochthoeca cinnamomeiventris		X	
Phyllomyias nigrocapillus		X	
Pipraeidea melanonota			X
Pipreola riefferii	X		
Poecilotriccus ruficeps		X	
Pogonotriccus ophthalmicus		X	
Pogonotriccus poecilotis		X	
Pyrrhomyias cinnamomea		X	
Rupicola peruviana	X		
Scytalopus latrans		X	
Scytalopus micropterus		X	
Sericossypha albocristata		X	
Synallaxis azarae		X	
Tangara cyanicollis	X		
Tangara labradorides		X	
Tangara nigroviridis		X	
Tangara parzudakii	X		
Tangara vassorii	X		
Tangara xanthocephala	X		
Thamnophilus unicolor		X	
Thraupis cyanocephala	X		
Thraupis palmarum	X		
Thryothorus euophrys		X	

Troglodytes solstitialis			X	
Turdus fuscater				X
Turdus serranus		X		
Vireo leucophrys			X	
Wilsonia canadensis			X	
Xiphocolaptes promeropirhynchus			X	
Xiphorhynchus triangularis			X	
Zimmerius chrysops			X	
Zonotrichia capensis				X
**3000**				
**Galliformes**				
Penelope barbata		X		
**Columbiformes**				
Columba fasciata		X		
**Apodiformes**				
Adelomyia melanogenys	X			
Aglaeactis cupripennis	X			
Boissonneaua matthewsii	X			
Chalcostigma herrani	X			
Coeligena iris	X			
Coeligena lutetiae	X			
Coeligena torquata	X			
Eriocnemis vestitus	X			
Heliangelus viola	X			
Lafresnaya lafresnayi	X			
Metallura odomae	X			
Metallura tyrianthina	X			
Pterophanes cyanopterus	X			
**Piciformes**				
Aulacorhynchus prasinus				X
Piculus rivolii			X	
**Passeriformes**				
Amblycercus holosericeus			X	
Ampelion rubrocristatus		X		
Anairetes parulus			X	
Anisognathus igniventris				X
Anisognathus lacrymosus		X		
Atlapetes latinuchus				X
Atlapetes pallidinucha				X
Basileuterus coronatus			X	
Basileuterus nigrocristatus			X	

Buarremon torquatus			X
Buthraupis montana	X		
Catamblyrhynchus diadema		X	
Catamenia homochroa			X
Chlorornis riefferii			X
Cinnycerthia unirufa		X	
Cnemoscopus rubrirostris		X	
Conirostrum albifrons		X	
Conirostrum cinereum			X
Conirostrum sitticolor			X
Cyanolyca turcosa		X	
Diglossa albilatera			X
Diglossa humeralis			X
Diglossa lafresnayii			X
Diglossopis cyanea		X	
Dubusia taeniata			X
Elaenia albiceps			X
Elaenia pallatangae			X
Grallaria nuchalis		X	
Grallaria ruficapilla		X	
Grallaria rufula		X	
Grallaria squamigera		X	
Grallaricula nana		X	
Hellmayrea gularis		X	
Hemispingus superciliaris		X	
Hemispingus verticalis		X	
Iridosornis rufivertex			X
Margarornis squamiger		X	
Mecocerculus stictopterus		X	
Myioborus melanocephalus		X	
Ochthoeca rufipectoralis		X	
Pheucticus chrysogaster			X
Phyllomyias nigrocapillus		X	
Pipreola arcuata	X		
Pseudocolaptes boissonneautii		X	
Scytalopus latrans		X	
Scytalopus parkeri		X	
Synallaxis azarae		X	
Tangara vassorii	X		
Thraupis cyanocephala	X		
Thryothorus euophrys		X	

Troglodytes solstitialis	X	
Turdus fuscater		X
Zonotrichia capensis		X
