# Supplementary material for: Spatio-temporal variation in bird assemblages is associated with fluctuations in temperature and precipitation along a tropical elevational gradient
Source: PLoS One. 2018 May 10;13(5):e0196179. doi: 10.1371/journal.pone.0196179 (PMC5945003; doi:10.1371/journal.pone.0196179)
Supplement: S3 Table — (PDF) [file pone.0196179.s007.pdf]

S3 Table. Variance and standard deviation (SD) of the random effects in the models testing the effect of elevation and season on bird a) abundance, b) evenness and c) species richness. Species richness model in d) also includes abundance as a fixed effect. See Table 2 in the main manuscript for model estimates of fixed effects.

	Random effect	Variance	SD
a) Abundance	Plot : site	0.08	0.27
	Site	0.02	0.14
	Sampling month	0.03	0.17
b) Evenness	Plot : site	<0.01	0.02
	Site	0.00	0.00
	Sampling month	0.00	0.00
c) Richness	Plot : site	0.03	0.18
	Site	<0.01	0.05
	Sampling month	0.01	0.11
d) Richness*	Plot : site	0.01	0.09
	Site	0.00	0.00
	Sampling month	0.00	0.00
