# Supplementary material for: Spatio-temporal variation in bird assemblages is associated with fluctuations in temperature and precipitation along a tropical elevational gradient
Source: PLoS One. 2018 May 10;13(5):e0196179. doi: 10.1371/journal.pone.0196179 (PMC5945003; doi:10.1371/journal.pone.0196179)
Supplement: S4 Table — (PDF) [file pone.0196179.s008.pdf]

S4 Table. Variance and standard deviation (SD) of the random effects in the models testing the effects of temperature, precipitation and resource availability on eight temporal replicate counts in a) bird abundance b) evenness and c) species richness at three elevations. See Table 3 in the main manuscript for model estimates of fixed effects.

a) Bird abundance	Random effect		Variance	SD
Temperature 3000 m	Plot: temperature	Intercept	0.34	0.58
		Temperature	0.10	0.32
Temperature 2000 m	Plot: temperature	Intercept	0.07	0.26
		Temperature	0.33	0.58
Temperature 1000 m	Plot: temperature	Intercept	0.17	0.41
		Temperature	0.22	0.47
Precipitation 3000 m	Plot: temperature	Intercept	0.25	0.50
		Precipitation	0.02	0.14
Precipitation 2000 m	Plot: temperature	Intercept	0.04	0.20
		Precipitation	0.01	0.11
Precipitation 1000 m	Plot: temperature	Intercept	0.03	0.17
		Precipitation	0.01	0.09
Resources 3000 m	Plot: temperature	Intercept	0.26	0.51
		Resources	0.01	0.08
Resources 2000 m	Plot: temperature	Intercept	0.04	0.20
		Resources	0.02	0.16
Resources 1000 m	Plot: temperature	Intercept	0.05	0.23
		Resources	<0.01	0.06
b) Bird evenness				
Temperature 3000 m	Plot: temperature	Intercept	<0.01	0.01
		Temperature	<0.01	0.04
Temperature 2000 m	Plot: temperature	Intercept	<0.01	0.01
		Temperature	0.03	0.16
Temperature 1000 m	Plot: temperature	Intercept	<0.01	0.01
		Temperature	<0.01	<0.01
Precipitation 3000 m	Plot: temperature	Intercept	<0.01	0.04
		Precipitation	<0.01	0.03
Precipitation 2000 m	Plot: temperature	Intercept	<0.01	0.01
		Precipitation	<0.01	0.01
Precipitation 1000 m	Plot: temperature	Intercept	<0.01	0.02
		Precipitation	<0.01	0.02

Resources 3000 m	Plot: temperature	Intercept	<0.01	0.03
		Resources	<0.01	0.01
Resources 2000 m	Plot: temperature	Intercept	<0.01	0.01
		Resources	<0.01	<0.01
Resources 1000 m	Plot: temperature	Intercept	<0.01	0.02
		Resources	<0.01	0.02
c) Bird richness				
Temperature 3000 m	Plot: temperature	Intercept	0.27	0.52
		Temperature	0.18	0.43
Temperature 2000 m	Plot: temperature	Intercept	0.05	0.22
		Temperature	0.71	0.84
Temperature 1000 m	Plot: temperature	Intercept	0.08	0.28
		Temperature	0.11	0.34
Precipitation 3000 m	Plot: temperature	Intercept	0.10	0.31
		Precipitation	<0.01	0.05
Precipitation 2000 m	Plot: temperature	Intercept	0.02	0.13
		Precipitation	0.03	0.17
Precipitation 1000 m	Plot: temperature	Intercept	0.01	0.09
		Precipitation	0.02	0.13
Resources 3000 m	Plot: temperature	Intercept	0.06	0.25
		Resources	<0.01	0.06
Resources 2000 m	Plot: temperature	Intercept	0.03	0.18
		Resources	<0.01	0.03
Resources 1000 m	Plot: temperature	Intercept	0.02	0.14
		Resources	<0.01	0.01
